# The Non-Combatants

**Published:** 1917-10

**Authors:** 


**A Monthly Review of Medicine and Surgery**

EDITOR AND PUBLISHER, DR. A. L. BENEDICT, 228 Summer St., corner of Elmwood Ave., Buffalo, (Address for all communications. Please make personal and telephone calls before 1 P. M.)

Third, in age, on the western continent. Independent, but supports professional organizations. News limited mainly to Western N. Y. and Penn.

Subscription $2.00 a year; $3.00 for two years in advance. Changes and errors in address or failure or duplication of delivery, should be reported immediately.

Yearly Volume 73 OCTOBER, 1917 Number 3

### The Non-Combatants.

It is said that the old prejudice against the medical officer is appearing in training camps. Why?—

Inferior mental material? 25% or more of the whole medical profession are college men. All have had the medical course which is as long and, except in discipline, as hard as that at West Point or Annapolis. Unless waived by authority, on account of immediate needs, candidates go through the same training in camp as other reserve officers.

Inferior physical material? There is not the proportion of rejections as for privates.

Lower standard of patriotism? The country expects to get, by draft, 4% of the total male population or 20% of that of the first decade of adult life. It expects to get by volunteering nearly 20% of the entire man strength of the medical profession and more than 100% of the age group mentioned.

Playing safe? Statistics of this war show that the mortality of officers is higher than of privates, of medical officers highest of all, of aviation officers lowest.

A Non-Combatant? Yes, technically by army regulations, the spirit of which has been repeatedly violated in the interests of all by army surgeons who have led troops that they could not command. On the whole, in direct authority over subordinate officers and privates in the medical corps, exposure to military hazards, etc., the medical department is more closely analogous to the line than any other special department. It is not expected that any officer shall normally engage in direct combat.

Efficiency? Conservation of one's own forces is a fundamental in modern military science. In the past, deaths from diseases outnumbered traumatic war deaths, from 10:1 down to 2:1 in the most favorable circumstances. The present war presents hygienic and sanitary handicaps never equaled before except on a small scale. Yet the deaths from disease and the disease incidence, wherever subject to the control of an efficient medical staff, have been, if anything, less than for the same number of men in peace conditions. No other branch of the service has either by conservation of its own forces or destruction of the enemy's, achieved anything like this result, offensive and defensive devices having, on the whole, neutralized each other.

Carried to its logical limit, the old prejudice against the army surgeon would result as follows: 1. A general destruction of discipline, the private who fires a gun being the only real soldier and combatant; 2. A contempt for camouflage, the mathematic accuracy of artillery and particularly the barrage, for engineering and for every specialized development of modern warfare; demoralization of all special staff departments and return to 18th century military conditions; 3. A return to the novel and picture-play conception of heroism, unless sacrifices of the individual for glory, waving flag, shouting Ee plooribuhs Yoon'm. This sort of heriosm is now rewarded by 5 days' imprisonment. Cheerful subjection to necessary risks is, of course, needed but the aim of every commander is to avoid useless waste of life of his troops.

